# Interprofessional identity: an ethnography of clinical simulation learning in New Zealand

**DOI:** 10.1186/s12909-021-03054-3

**Published:** 2022-01-21

**Authors:** Pauline Cooper-Ioelu, Tanisha Jowsey

**Affiliations:** 1 Learning and Teaching Unit, Faculty of Medical and Health Sciences, Room 327, Building 505, Level 3, 85 Park Road. Grafton, Auckland, 1023 New Zealand; 2 Centre for Medical and Health Sciences Education, Building 507, Level 2, Room 2024, 28 Park Ave, Grafton, Auckland, 1023 New Zealand

**Keywords:** Interprofessional education, Liminality, Health professions, Pre-registration training, Ethnography

## Abstract

**Background:**

This article explores the experiences of clinical healthcare students on an interprofessional simulation course in Auckland, New Zealand. The four-day course aims to provide a formative learning experience for final year medical, pharmacy, nursing, and paramedicine students. It focuses on building skills in professionalism, communication, leadership and interprofessional safe teamwork through structured learning activities and clinical simulation scenarios.

**Methods:**

In 2018, we commenced focused ethnographic research involving participant observation, field notes, interviews, photography and ethnographic film.

**Results:**

A total of 112 students participated in this research from the disciplines of medicine (*n* = 53), nursing (*n* = 27), pharmacy (*n* = 17), and paramedicine (*n* = 15). In a revisit to Van Gennep’s (1972) seminal work on liminality, we suggest that the course represents a liminal space where students’ ideas about what it means to be a healthcare ‘professional’ are challenged, disrupted and reconstructed. We observed students emerging from the course with transformed professional and interprofessional identities.

**Conclusions:**

We posit that the ritualised and liminal nature of the course plays a role in the development of interprofessional identities by interrupting the reproduction of siloed biomedical culture. Students are challenged to become effective team members alongside other students and experts from other professions. We discuss these findings as they relate to medical and health sciences education.

## Introduction

Internationally, interprofessional education (IPE) is a staple of many medical and health professions curricula; it is embedded within educational programmes and is present throughout the continuing education of healthcare professions [1–3]. The Centre for the Advancement of Interprofessional Education (CAIPE) defines interprofessional education as occurring “when two or more professions learn with, from and about each other to improve collaboration and the quality of care” [4]. IPE is an effective means of enabling practitioners to understand each other better, work more collaboratively, and improve patient care and service delivery [5, 6].

One key idea that is connected to IPE is the idea of professional socialization. This can be defined as “the process of learning values, attitudes, norms, and behaviors of professional roles” [7]. Health professional education has traditionally focused on training students to become highly committed and loyal to their profession and to internalise a strong sense of belonging. While health professionals may work alongside each other they do not necessarily work in purposeful collaboration [8]. This professional introspection and the subsequent development of identities that align with one’s profession can be historically located. In the latter half of the twentieth century, as the professions developed and specialised, they battled to define their professional roles and scope of practice, often in competition with the other professions [9, 10] Many health professionals preferred to see themselves as different and even sometimes as better than other professions. Early socialization fostered a strong *uniprofessional* sense of the self that reproduced hierarchies of practice and stereotypical understandings of other professions [10].

It has been suggested that the inclusion of IPE strategies early in health professional programs is an important strategy to address stereotypes and professional tensions that impact future interprofessional collaboration [11]. Many studies also attest to the power of IPE in clinical training (such as [12, 13]) and the need for early socialization to encourage the development of an interprofessional aspect to professional identity [14]. However, few studies have explored the relationship between IPE and the formation and construction of *interprofessional* identities - in-situ *-* in the pre-registration stages of clinical training [2, 15].

A recent literature review concerning professional and interprofessional identities found that existing studies define interprofessional identity poorly and that few studies have attempted to measure interprofessional identity formation [16]. In their Extended Professional Identity Theory, Reinders et al. [17] explain, “an interprofessional identity is a social identity based on a widening circle of group membership that consists of more than one profession (p .1).” The Extended Professional Identity Theory consists of three interrelated characteristics: interprofessional belonging, interprofessional commitment and interprofessional beliefs (p. 2). Khalili and colleagues [14] describe interprofessional identity as a dual identity that can be developed alongside an uniprofessional identity through processes of socialization. The development of an interprofessional identity results in a two-fold identification with one’s profession and the interprofessional community. These authors suggest that IPE literature has focused predominantly on describing IPE programme development and changes in learner attitudes, knowledge and skills following IPE experiences. Khalili et al. [14] suggest that IPE focus should be broadened to include socialization to better understand how a uniprofessional identity can be reshaped to include an interprofessional identity.

In this article, we suggest that an ethnographic approach may shed further light on activities that contribute to the development of an interprofessional identity. A focused ethnographic approach was used in this study of students on an interprofessional simulation course because it allows researchers to look at students in a particular cultural setting and observational methods allow for greater flexibility than self-reporting methods alone. In this focused ethnography, we explore student experiences on a four-day pre-registration simulation IPE course. Our research question was: does the course inform students’ sense of professional identity, and if so, in what ways? The ethnographic method allowed us to simultaneously observe contextual issues alongside how students made sense of their developing professional identities during the course.

## Background

### Context of the course

In Auckland, three tertiary institutions have partnered to run an interprofessional simulation training course called Urgent and Immediate Patient Care Week (UIPCW). Medical, nursing, pharmacy and paramedicine students are required to attend the course during the final year of their degree. The same course is run seven times a year so that each student can participate once and a cohort size of approximately 80 students can be maintained. Students work in interprofessional teams in a range of educational environments during the course, moving from classroom activities to high fidelity simulation scenarios. Learners attempt to apply the knowledge and skills they have acquired during their professional degrees. All students have had some clinical experience during their studies. Students are encouraged to critically reflect on clinical decision-making; what they know and do not know, what it means to be professional, and their scope of practice.

Students are asked to reflect actively with peers and faculty from other professions. Teaching staff facilitate both classroom learning and the pre-briefings, simulations, and debriefing of the simulations. To prepare, teachers attend a training day where they are briefed on the learning outcomes, the course content, and simulations. Learners arrive with little formal training on communicating effectively with their peers from other professions or working effectively in interprofessional teams. Students from the University of Auckland come to the course with some experience of learning in interprofessional teams. Experiences in previous years of their curriculum include during three short courses: Population Health Intensive (five days), Quality and Safety (two days), and Māori Health Intensive (five days). While these other courses offer students important interprofessional socialization learning, UIPCW offers students their first interprofessional *simulation* experience where they can embed their interprofessional learning through practice.

### Professional identity and clinical cultures

The transition from student to healthcare professional is a complex humanistic journey. It takes many years of clinical practice and metacognition to develop the expert knowledge, self-regulation and fiduciary responsibility required to perform as an accomplished clinician [18]. Professionalism in healthcare includes the presence of essential qualities such as caring for, respecting, and being responsive to the needs of patients; acting honestly and ethically; working in partnership with patients and colleagues; and to maintain and improve standards [3]. Students learn about professionalism through formal curricula, role modelling, the hidden curriculum, policies and procedures, and trial and error. Hafferty [19] explains that “considerable learning (some think most) takes place outside of the domain of the formal curriculum and that such learning involves indoctrination in the unwritten rules of studenthood and medical practice” (p. 2151).

Health professions education literature acknowledges that becoming a health professional involves more than simply attaining technical skills and mastery of biological knowledge [20]. As trainees, professionals learn norms and ways of acting that are acceptable within their professional disciplines [14, 19]. Drawing on social identity theory, Khalili et al. [14] argue that an “individual’s identification with a social group (that is a specific profession) results in a profession-specific cognitive map and a system of orientation toward one’s chosen profession” which results in “in-group favoritism that creates high levels of trust and cohesiveness among professional members and out-group discriminatory bias that leads to distrust toward those outside of their group” (p.1469). This process of professional socialization commences well before students have even stepped into a lecturer theatre and it continues as they move from trainee spaces as registered health professionals.

Other discourses concerning healthcare identity formation suggest that socialization occurs through the reproduction of biomedical culture [21]. Foucault’s *The Birth of the Clinic* [22] provides much of the theoretical foundations that underpin the concept of biomedicine. Foucault coined the term ‘medical gaze’ to describe the condition whereby clinicians are enculturated into seeing the person through an objective lens as a body, made up of identifiable and measurable separate parts. Historically, from early in the clinical enculturation process, students are taught – albeit implicitly via the hidden curriculum – to prioritize ‘competence’ over caring, giving priority to fixing ailments, and not necessarily the holistic wellbeing of their patients [23]. Professional schools have implemented many creative initiatives to combat this ‘gazing’ including development of compassion and empathy, communication, and professional skills.

### Becoming professional in liminal spaces

The concept of liminality adds some richness to understanding the processes of socialization in pre-registration training of health professionals. Van Gennep [24] describes the process of liminality as being one of transformation from one state of being to another. It is usually unidirectional and is marked socially through rituals. Rituals are a series of actions performed according to a prescribed order, often – but not necessarily – including religious or spiritual elements (we will return to rituals below). Taylor [25] describes liminals as “threshold people” at a juncture of changing their professional status. Hopeful initiates reproduce what they have been taught and what they have learned through their own ritualised experiences. These rituals make unseen structures temporarily physically visible [20]. In liminal spaces, shifts in identity are in a constant state of flux as the self is “not something ready-made, but something in continuous formation” ([26], p. 235). Ever-changing discourses inform the social self in new ways; an individual’s concept of self evolves, strengthens, and matures through professional education and experience [27].

**Fig. 1:**
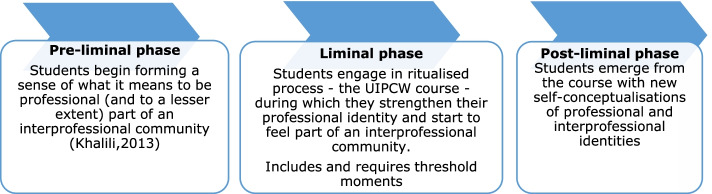
Van Gennep’s process of liminality applied to student inter-professional identity formation during the UIPCW course

In liminal spaces, students are transformed from one state of being to another and, in a sense, are suspended between the worlds of the classroom and the clinic: students are not yet certified professionals, but neither can they be considered to be lay. The label of “student-doctor” or “student-nurse” speaks to a suspended and ambiguous state. These terms designate an endpoint, but they also symbolise “being at once no longer classified as one thing (student) and being not yet classified as another thing (doctor/ nurse)” ([28], p. 109). This transformation state entails a reformulation of the learner’s frame of meaning and an accompanying shift in the learner’s subjectivity. Accordingly, liminality requires a constant re-authoring of self; learners are perpetually acquiring, using, and internalising new forms of written and spoken discourse to reform who they are and aspire to be (see Fig. 1).

### Becoming interprofessional

Barr [6] defines interprofessional practice (IP) as the collaboration of members of two or more different professions. IP can strengthen relationships between colleagues across various professions, departments and disciplines, improve staff morale and retention, and contribute to a positive learning environment. Effective IP can also enhance patient safety [12, 29] . In New Zealand, there is no unified framework for IP, although many of the health professions include IP in their individual professional standards [30–32]. The Interprofessional Education Collaborative (IPEC) [33] has developed four core interprofessional competencies that are useful to conceptualise IP. These are 1) teamwork and team-based practice, 2) interprofessional communication practices, 3) roles and responsibilities for collaborative practice, and 4) values and ethics for professional practice.

Educational and interprofessional experiences such as UIPCW challenge students to construct and reconstruct meaning about their professional identity [34]. Certain concepts, practices, or learning experiences can act as a portal through which new perspectives open up for the learner. Learners may also experience difficulty letting go of previously-held ideas, beliefs or knowledge. Threshold concepts – difficult or troublesome knowledge - can act as rites of passage in liminal spaces that permit new and previously inaccessible ways of thinking and practicing and set off a change to the personal, professional and social self [35, 36]. As students transition through the liminal phase, the student reaches a new threshold where previously-held ways of thinking and being can no longer be sustained [36]. The student emerges (post-liminal) with new-found ways of thinking and practising. In contrast to later periods in a professional’s career, the liminal student self is more malleable [25, 28].

To illustrate in the *transformational* elements of IP rites of passage, student trainees may initially seek to copy the conventions and practices that they have observed in their teachers and clinicians from their own profession – they draw on skills from a uniprofessional toolbox. Yet with exposure to interprofessional practices, these ways of being and acting are disrupted. Students are forced to reconsider a siloed approach to care, and consider new understandings of what caring for a person means and their own role as a future healthcare professional. Students learn about the scopes of practices of other professions but also the relationship between their own scope of practice in relation to other groups. In this way, students are forced to shift their understandings and practices to a more collaborative and collegial approach to care.

We detail how students undergo liminal processes that contribute to the construction of interprofessional identities during the UIPCW simulation programme. While activities are structured around commonplace clinical encounters, simulation and debriefing sessions challenge students to view their decisions from the perspectives of students and staff with different scopes of practice to themselves. Students’ conceptions of their professional identity evolve and adapt, and an interprofessional identity emerges.

## Method

A critical ethnographic approach underpins this methodology to illuminate the values, meanings and scopes of practice informing student experiences [37–40].

### Ethics

Ethics approval for this research was obtained from the University of Auckland Human Participants Ethics Committee. Reference Number 020759.

### Setting

Since 2013, the University of Auckland has run an interprofessional simulation-based course, which is now a mandatory course for all fifth-year medical students, fourth-year pharmacy students, and third-year nursing students. The University of Auckland has partnered with the Auckland University of Technology to include staff and students from the Department of Paramedicine in UIPCW. Nursing students from Manukau Institute of Technology also participate in UIPCW. The course runs seven times across the academic year as a consecutive four-day course (22 contact hours) (except during the Covid-19 pandemic in 2020 and 2021).

Before attending the course, students are allocated to mixed professional groups of eight to eleven members. Each of the four days has a “theme”; community care, palliative care, and the two remaining days focus on in-patient ward care. Students attend the full four days except for paramedicine students who attend only the “community” and “palliative care” days. A mixed professional team of students (with at least one student from each profession) are selected to participate in each simulated scenario; all students participate in at least one scenario per day. Students who are not participating in the simulation are tasked with active observation. Simulations are structured around various scenarios depending on the day’s theme and involve both patient actors and computerised simulation manikins (Laerdal 3G SimMan).

The learning outcomes for all students at UIPCW are: to describe the roles and expertise of own and other health disciplines and to recognise the limits of expertise; to practice information sharing; and to practice using structured communication tools. Students must also perform systematic assessment of an acutely unstable patient and initiate management within their scope of practice.

While all students have experienced clinical work in some capacity, UIPCW puts students in the driving seat – they make the decisions, and the simulation is primarily informed by the decisions made by students in the room. The simulations are designed to hone clinical skills, cognitive thinking, and communication in an interprofessional context. Before the simulation, students attend a pre-briefing – usually facilitated by two instructors from different disciplinary backgrounds – where they receive the scenario and any relevant documentation and information. Once the simulation has ended, all students attend a structured debriefing session where students and facilitators tease out the details of the case, and student decision-making is analysed. Students are encouraged to reflect on their performance within their scope of practice and in relation to their peers’.

### Participant recruitment

**Fig. 2:**
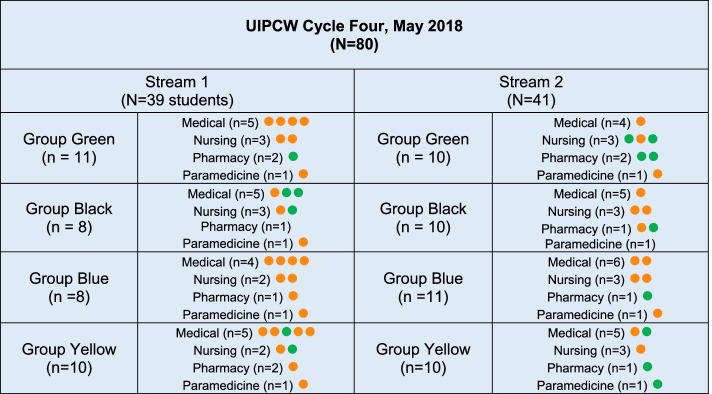
Students were grouped into two streams. Orange (film) and green (not-to-be-filmed) stickers used by participants

All students and faculty participating in the UICPW during two cycles in May 2018 were invited to participate. The academic director of each discipline sent out an email alerting students and staff to the research before they commenced the course rotation. Upon arrival, staff and students were provided with a participant information sheet and consent form. Some chose to participate, and others declined (participant details are in Table 1 and Fig. 2).

**Table 1:** Participants

*Research Participants*	*Cycle Three*		*Cycle 4*	
	*May 8–11, 2018*		*May 22–25, 2018 (filming)*	
	*N = 113*		*N = 123*	
***Students***	*Attended*	*Consented to be observed*	*Attended*	*Consented to be observed*
*Medical*	*38*	*31*	*39*	*22*
*Nursing*	*16*	*11*	*22*	*16*
*Paramedicine*	*8*	*8*	*8*	*7*
*Pharmacy*	*11*	*8*	*11*	*9*
***Total Students***	***73***	***58***	***80***	***54***
***Staff***				
*Medical*	*14*	*14*	*16*	*16*
*Nursing*	*4*	*3*	*5*	*3*
*Paramedicine*	*3*	*3*	*5*	*5*
*Pharmacy*	*7*	*7*	*7*	*7*
*Radiography*	*1*	*1*	*0*	*0*
*Medical Education*	*2*	*2*	*0*	*0*
*Simulation Technician*	*3*	*3*	*4*	*4*
*Timekeeper/ researcher*	*2*	*0*	*2*	*2*
*Actor Patient*	*4*	*4*	*4*	*4*
***Total staff***	***40***	***37***	***43***	***41***

### Participation and non-participation issues

In the consent forms, students and staff were asked to tick a box to consent to participate in the research and tick a second box if they also consented to be filmed. The film consent details followed film industry-standard consent protocol. The consent form had two boxes for students to consider ticking; the first was to participate in research but not be filmed, and the second was to participate and be filmed. In contrast to most scientific human research, the consent form clarified that participants would be identifiable in the film. Participants had two weeks from the time of filming to withdraw their consent. After that time, editing would commence and the director would own the audio-visual material and, as such, would have the right to edit and distribute the film content.

**Fig. 3:**
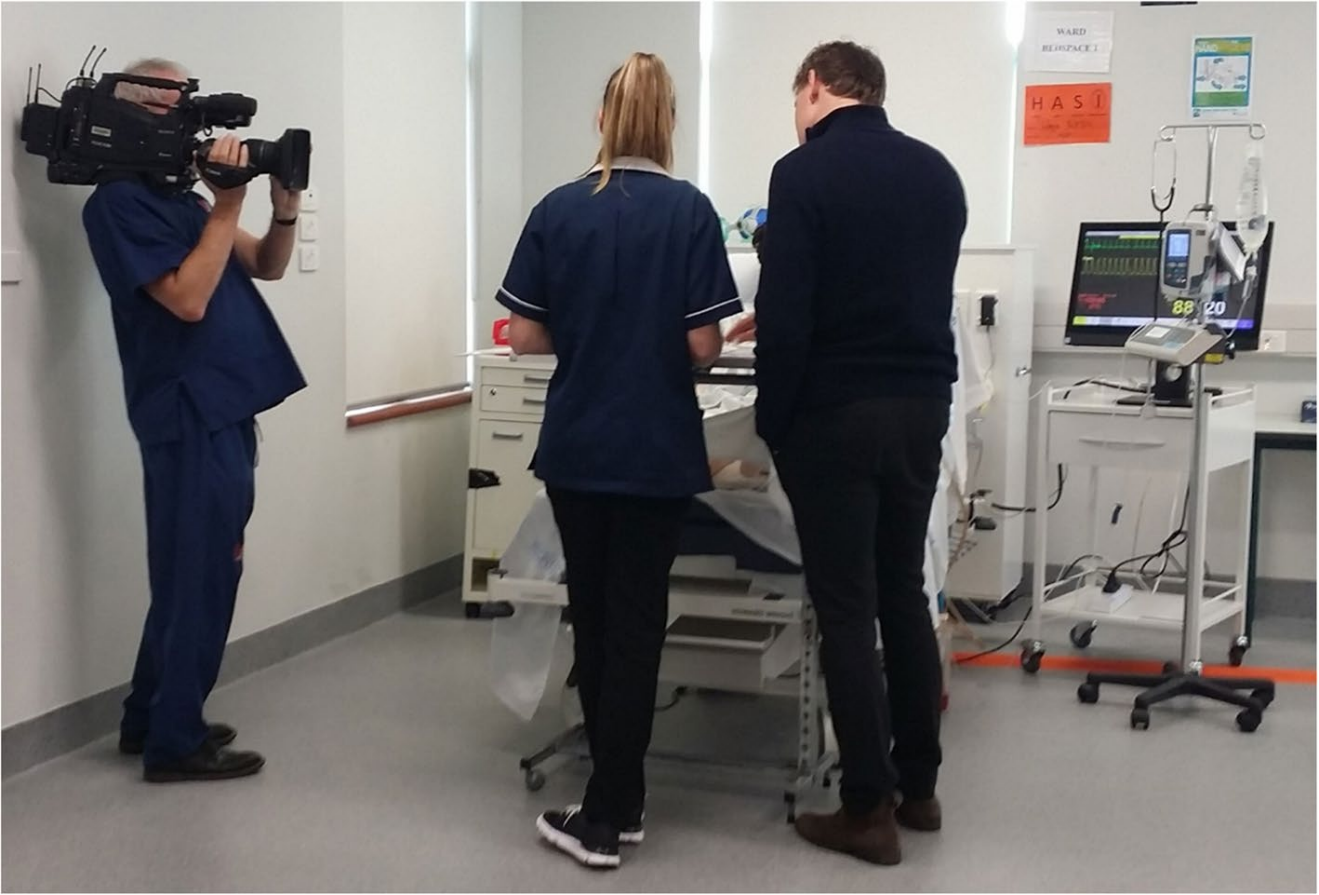
Cameraman filming participants who consented to be filmed during simulation scenario of acutely unwell deteriorating patient. The image was taken with permission by Tanisha Jowsey, May 2018

All students were placed in groups for the duration of the course. Students who chose to take part were grouped together. Film cameras were set up to only include participants. When non-participants entered the shots, we moved the camera. In situations where there was insufficient time to move the camera - or it was deemed distracting to students to move the camera - we left it in place and later blurred out identifying features of non-participants. We tried to minimise opportunities for the research to interfere with the course or student learning for both participants and non-participants and we did this by setting up stationary cameras in the background where possible, though in some simulations the camera man (an enormous film industry camera) was close by to participants during their scenarios (see Fig. 3).

Students who ticked the first box but not the second box were given a green sticker to wear on their name badge. Students who also ticked the second box were given an orange sticker to wear on their name badges. Students who chose not to participate did not wear a sticker. The purpose of the stickers was to make it clear to the film and research crew the capacity in which students were participating.

### Ethnographic study design

We used an ethnographic approach [38, 41–43] to explore the social lived experiences of staff and students during UIPCW through participant observation, field notes, photographs, observational film and recorded interviews with research participants. We utilised a focused ethnographic approach [44], which involves researchers being immersed – intensively – in the research field over a short time and collecting different data types. Part of the research method involved making an ethnographic film called *Prepared to Care*, which we describe elsewhere [45, 46]. It is freely available on YouTube (https://youtu.be/467bAuCAv1E) and Vimeo (https://vimeo.com/410866046).

### Positionality and reflexivity

The lead researcher (second author) had contributed to teaching and research for UIPCW for three years before undertaking this research. So, although the method is focused (time-compressed) ethnography, it is also informed by her long-term familiarity and immersion with the course [44]. The first author’s background is in the social sciences and clinical education, and she had no previous exposure to UIPCW before the research. The second author’s background is in anthropology and clinical education. She had previously been involved in evaluating student learning on UIPCW and came to this research with a positive view of UIPCW as an effective course. Although both authors teach undergraduate students, we ensured that students on UIPCW were not students we had previously taught in other contexts.

### Analysis

We undertook an iterative process of analysis [47]. Four members of the research team [TJ, M-J, PL, P-CI) collated, summarised and thematically coded the observational written data (not including film data) by hand [47]. Similar codes were grouped together to create higher-order themes. We checked these concepts back against the data regularly to ensure that interpretation was consistent. We read and re-read the dataset to ensure data immersion. At the same time as this analysis was underway, one member of the research team [TJ] was editing the ethnographic film output of this study, which added an audio-visual mode of data immersion. Another research team member [PC-I] also viewed the film data, including recorded interviews. The research team used observational film and recorded interviews to triangulate the data collected during field participant observations. This in-depth process of data immersion and coding allowed the team to agree on emergent themes, which formed the basis for the ethnographic engagement with existing social theory.

## Results

**Fig. 4:**
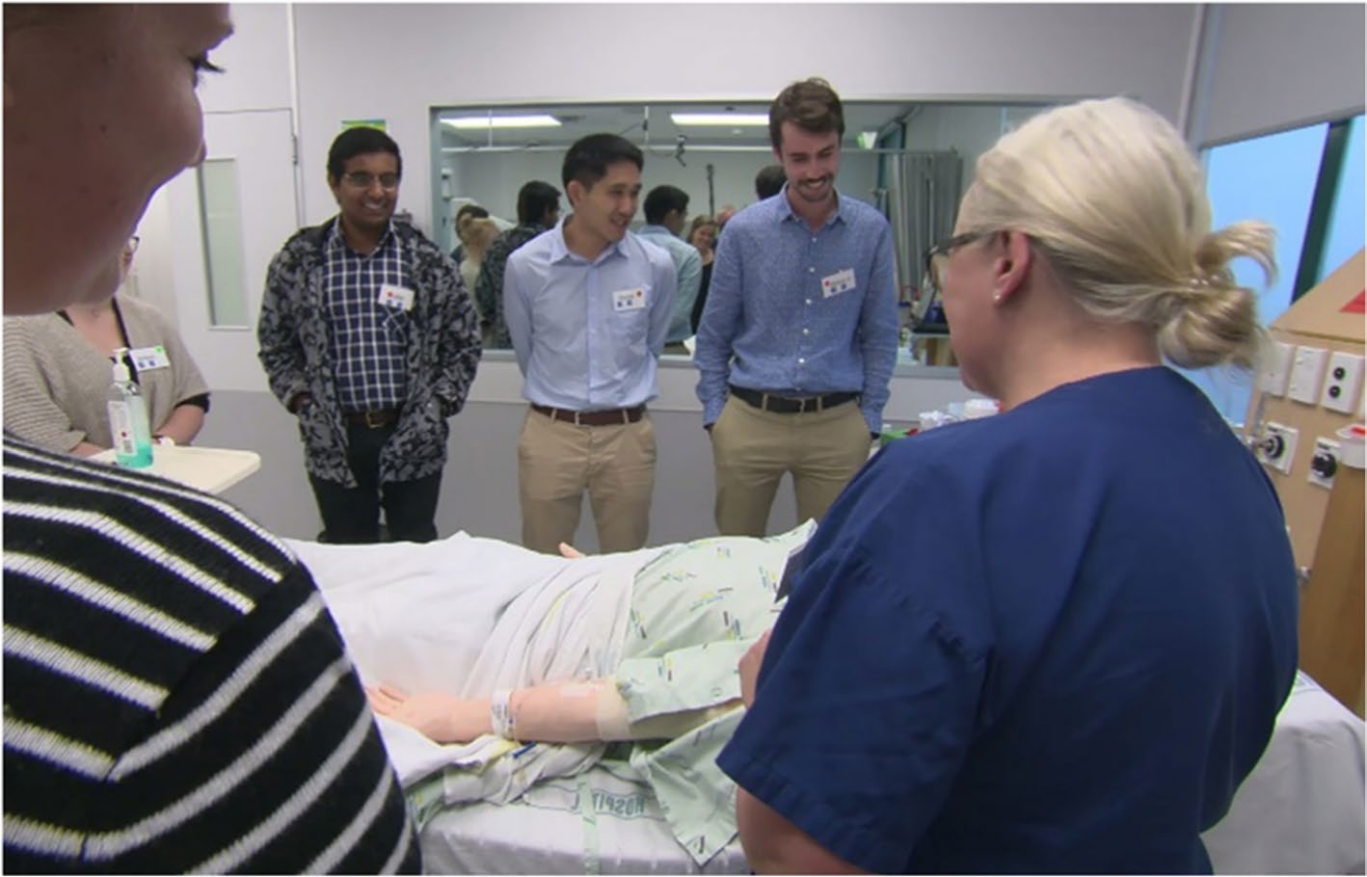
Staff orient students to simulation environment prior to the scenario commencing. The photo was taken with permission by Tanisha Jowsey, May 2018

Of 153 students who attended UIPCW in May 2018, 112 consented to participate in this research from the disciplines of medicine (*n* = 53), nursing (*n* = 27), pharmacy (*n* = 17), and paramedicine (*n* = 15) (see Table 1). Slightly more students from cycle three (58/73 = 79%) consented to participate than from cycle four (54/80 = 71%). Eighty-seven staff were invited to participate, and of these, 84 consented (see Figs. 2 and 4).

### Becoming

As part of the analysis process, we noticed ‘becoming’ as a higher-order concept. In the context of health professions education, the concept of becoming describes the multifaceted process by which students transition from laypersons to professionals [48]. We define it here based on how it epitomises the data.

*Becoming (verb):*

Emergent trust in self and other health care professionals. The individual develops knowledge and embraces unknown situations, spaces, bodies, people, and moments. They let go of prior thoughts, practices, and experiences and embrace new ways of thinking, practicing, and being *with* others.

The process of *becoming* was evident in the way participants experienced the course. During UIPCW, we observed notions of what it means to be professional in a constant state of movement. Students experienced tensions of the past and present *professional* self; they were confronted with new collegial realities of practice that promoted new ways of being and thinking. Students also constructed their identity in relation to other disciplinary groups and others’ scope of practice; working interprofessionally prompted students to deeply reflect on their relationship to others in the disciplinary team and construct their identity in *relation* to other professional groups.

The concept of liminality has explanatory power when it is considered alongside the concept of becoming, specifically how students construct their identity in the “betwixt and between” [24, 49] state as liminal people. The liminal space is characterised by fluidity and ambiguity and requires the subject to re-evaluate oneself constantly [50]. In liminal spaces we see identities shift rapidly and with relative ease. By employing focused ethnographic methods, we observed changes to the professional and interprofessional self over time, in situ. We outline four emergent themes that connect to the concept of *becoming*: scopes of practice, hierarchies of power, the biomedical gaze, and interprofessional identity.

### Scopes of practice

One area where students demonstrated their processes of becoming professional was through their emergent understandings of their own scope of practice and that of others. Scope of practice refers to the duties, roles and purviews associated with a specific profession. A nurse’s scope of practice is different to that of a dietician or a doctor. Students arrived at the course with various ideas about their own professional scope of practice, but they knew little about the scopes of practice of other professions. They demonstrated uncertainty and reluctance about accidentally underperforming at the boundary of their profession, or worse, over-stepping it. As the course progressed, students displayed increased confidence levels to act and contribute to the team and a heightened awareness of where these boundaries lay. For example, in one simulation of an acutely unwell patient, we observed a nursing student using the ISBAR communication tool (a ritual) competently. In the debriefing session (another ritual), the medical student (who entered the room slightly after others in the team) commented that they “felt supported” by the team and they felt confident because of the “good handover using ISBAR that included a very clear recommendation.”

On the first day of the course, students from all participating professions demonstrated low confidence during debriefing sessions and uncertainty in determining their own role in relation to others in the team. One medical student, for instance, was surprised when they “checked with the nurse and realized they have pieces of information that I did not.” This experience during the simulation forced the student to reassess their own role as ‘expert’ and reconceptualize the role of the nurse as a valuable team member and resource when diagnosing and treating a patient. In other situations, preconceived notions of the different professional roles resulted in ineffective care and uncertainly about how to perform. In a simulation scenario concerning asthma, a paramedicine student commented that she did not want to examine the throat area because she “didn’t want to rain on the parade” of the medical student in her team. This student regularly performed this examination during her clinical training and likely had more experience doing so than the medical student did.

We also frequently observed situations where medical, nursing and paramedicine students largely ignored the pharmacy student, mainly due to a lack of understanding about pharmacy roles in an acute setting. Students from medicine, paramedicine, and nursing all highlighted that they had never worked with pharmacy students. In almost all simulations observed on days one and two, medical students entered the scenario and largely ignored the pharmacy student, which they later explained (during the debriefing sessions) was due to unfamiliarity about what the pharmacy student could contribute. Unsurprisingly, pharmacy students reported feeling under-utilised and even displaced. A pharmacy student commented, “you are kind of just an extra pair of hands in that setting.” During a debrief, a paramedicine student asked a pharmacy student in her team, “what sort of information should I give you? I haven’t worked with a pharmacist before.” This conversation led to a broader understanding among the students of their various scopes of practice.

Students and faculty from other professions were impressed by the high confidence levels of the paramedicine students, yet they too experienced some difficulty discerning their role in an interprofessional team. Over lunch on the third day, a group of medical students approached a staff member, and one asked, “[w]e were wondering why we don’t get more learning like this because it feels like the paramedicine students have heaps of acute experience and know what to do.” Nevertheless, in team situations, paramedicine students experienced similar threshold moments about scopes of practice and roles. Reflecting on her experience after a simulation, one paramedicine student commented, “I started delegating tasks … and then sort of, towards the end it was like ‘oh crap, do these guys even know how to do this? Have I just asked these guys to do a task that they have no idea how to do?”

Part of this journey in identifying scopes of practice involved attending to students’ own assumptions and biases, often in the context of existing hierarchies of power. Students were required to reflect on their assumptions about other professional groups in structured handovers when their colleagues joined them in the simulation scenario. As the week progressed, we observed increasing mutual respect and a growing sense of collegiality between the professional groups, both in formal learning settings and during free time. We observed handovers that included more detailed relevant information according to the information needs of the handover recipient, demonstrating a newfound awareness of the importance of thinking and practicing interprofessionally.

As the course progressed, and as students became more aware of gaps in their knowledge about other professions, spontaneous conversations between the professional groups increased. We observed the emergence of mutual respect and a budding sense of collegiality and comradery in all learning settings (small group activities, simulations and debriefings and in the observation room). Students took greater care in handover and more quickly assembled into interprofessional groups. A simulation that we observed on day three of the course highlights this growing comradery well. All students (besides those participating in the simulation) entered the observation room to watch. One medical student observer asked a nursing student, “when and why you would have two nurses in the room to attend to a patient?” Meanwhile, in another corner of the room, a nurse and medical student talked about a possible diagnosis. In the simulation, the nursing student offered a thorough handover as the medical student entered the room. The medical and nursing students – both in the scenario and in the observation room – worked interprofessionally to create a shared mental model of diagnosis and appropriate treatment.

**Fig. 5:**
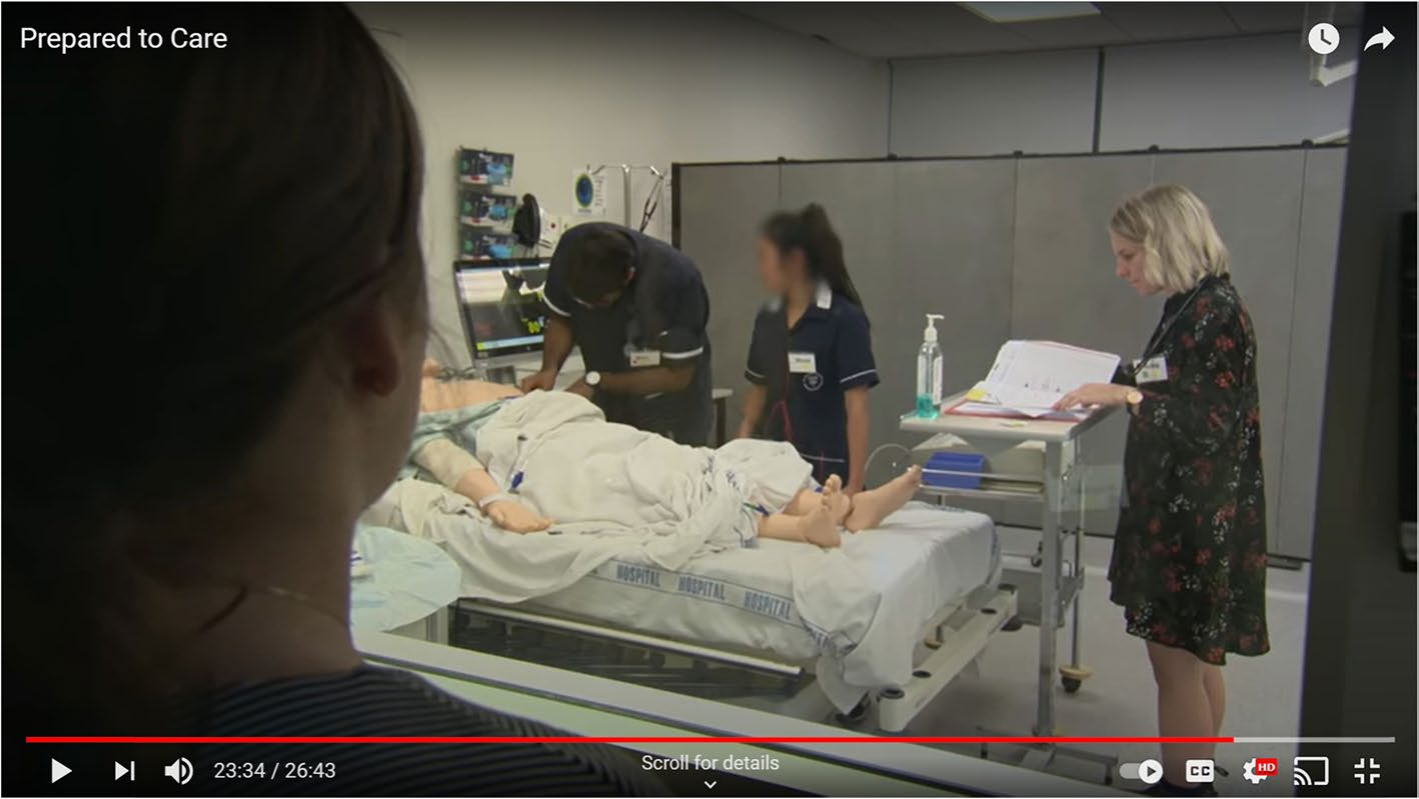
A medical student participant describes valuing interprofessional teamwork. Image is taken from the film Prepared to Care [23:34]

In addition, as students learned more about pharmacy scopes of practice, they became better at acknowledging and drawing on the pharmacy student’s specialised knowledge. In one debriefing session, for instance, a medical student stated, “there was a pharmacist in there, and I got good advice from her.” She commented that she was very grateful that the pharmacist was in the room to check the patients’ medication (see Fig. 5).

### Unravelling hierarchies of power

We observed that even in the relatively short duration of the course, the interprofessional nature of UIPCW (both in terms of faculty and student participants) unravelled assumptions concerning power and hierarchy in the clinical setting, particularly around conceptualisations of the role of doctors as leaders (i.e. in charge or leading decision making). Initially, we observed that pharmacy, nursing, and paramedicine students were reluctant to act on their intuition and instead looked to their medical peers for guidance and action in all learning spaces. Many students made assumptions about the ‘doctor’s’ role, particularly at the beginning of the week when medical students tended to voice their opinions and report back confidently following small group learning activities. It was also the case in simulation, observation, and debriefing activities. Non-medical students who were highly confident in their practice made remarks that spoke to the doctor’s role as leader and decision-maker in the clinical setting. One high performing paramedicine student who was happy to delegate and take control of an emergency situation remarked that “in a ‘real’ situation with a ‘real’ doctor…[I] would have just stepped back and observed.” Other confident students were doubtful of their ability in the presence of a medical student. One nursing student commented, “we feel as though a doctor knows more than us.” It did not matter that all students attending UIPCW were at similar places in their clinical training.

As the week progressed, we observed a flattening of the hierarchy. One nursing student commented that what she appreciated about UIPCW was that “everyone is on the same playing field rather than being in a hospital where you might not say something because there are certain levels.” A pharmacy student reported that she was skilled in doing particular tasks – such as preparing adrenalin – but she had not told the medical student this during the simulation, and the medical student had assumed the contrary. Following the debrief, the pharmacy student indicated that assumptions and core beliefs about hierarchy had gotten in the way of providing the best care for the patient. The lesson she took from the debrief was the importance of speaking up to promoted team efficacy and the best patient outcomes. She commented that she was determined to change her future practice towards sharing information no matter what the cost – she identified that she needed to let go of her previously–held ideas concerning hierarchies and teamwork.

### Disrupting the biomedical gaze

Conventional assumptions about the doctor’s role were unraveled by the medical students themselves when they displayed behaviors that broke from the traditional gazing mentality [51]. In a palliative care simulation, a medical student was presented with an upset patient. This student knelt next to the patient, was empathetic, put his hand on the patient’s shoulder and comforted the patient when they broke down in tears. In the observations room, students were in awe of the medical student’s bedside manner. Students commented, “he’s amazing with the patient”, and were surprised with the positive response it elicited from the patient. One student commented, “I have never seen a doctor kneel down before.” Medical students also learned from these examples of good practice. In the debriefing session, another medical student in the group remarked, “[it is] nice to see the empathetic side come forward – we don’t see that a lot.”

The interprofessional nature of the teaching broke down assumptions about what it means to care for a person. Clinicians and teaching staff from each profession contributed to the teaching of the course. Additionally, staff from other disciplines, including medical education and the humanities, contributed. The structured debriefing sessions were key interprofessional learning moments for students as these were facilitated by two staff members, usually from different health disciplines. Students’ preconceived beliefs about healthcare and roles were regularly disrupted in these sessions, and students were challenged to form new ways of viewing their profession and self as a trainee.

In one particularly stressful simulation where the patient wanted to make a complaint due to a medication error, students were discouraged from blaming the doctor solely. The facilitator stated, “[i]t’s not just the doctor’s role to apologize, it’s the team’s [role].” Students reported their take-home messages from that session as, “listen to the patient when they tell you something, even if it doesn’t fit your ideas,” and “mistakes don’t come from a single person.” This unravelling occurred under less stressful conditions also. We observed a particularly effective teacher, thinking out loud with the students in the observation room. She prompted the students to pay attention to how well the nurse was doing – “her emphasis on care…putting the bed up…the deep breathing techniques…the bucket next to the bed.” If the facilitator had opted to stay silent, these little details emphasizing the high quality of care for the person could have easily gone unnoticed. In another example, one medical student questioned if she focused too much on the patient’s emotional needs (as she was used to focusing more on medical needs). The facilitator promptly reassured the student that she was right to focus on the patient’s emotional needs.

The palliative care segment of the course had a special role in teaching many of the communication skills and professional attributes associated with empathetic and person-centred healthcare (these were often referred to by teachers and students as ‘soft’ or ‘core’ skills). One facilitator from paramedicine commenced his session with, “I’ve asked the palliative care team if there is anything they would like me to communicate to you today and every single one of them said this is a conversation…when we look at people, we often look at them as an illness, but patients don’t see themselves as an illness.” A nurse in attendance also contributed. She remarked, “no one remembers you for the science…they remember you for how nice you are and how well you control pain.” Another facilitator who was a palliative care nurse stated during the same session, “it can be little and big… pictures… …keeping feet warm, for some people that is really important” and “….the science is easy, the art is hard.” We observed many facilitators encourage students to move from a patient-centered to a person-centered approach to healthcare [52]; reoccurring themes included avoiding medical jargon, involving family, acknowledging the patient’s emotional needs, asking for help and letting the patient lead.

### Interprofessional identity

As the week progressed, we observed the development of students’ interprofessional identities. Students’ conceptions of self-evolved from a singular-profession view (for example, ‘I am training to be a nurse’) to an interprofessional one (‘I am training to be a healthcare professional’). This subtle transition was evident in such actions as students openly raising their concerns to peers regardless of their profession and the associated preconceived notions of hierarchy. On the final day of UIPCW, students had developed relationships of comradery and spoke to the value of their interprofessional learning, saying “we are all in this together” and “we are not alone” in this process of becoming a healthcare professional. A nursing student – when asked about her experience of the course – remarked:

One of the more intense things I wasn’t expecting was during the feedback session to be able to give positive critiques to other students. Because you want to suggest something for further learning, but you also don’t want to be bringing them down because they have actually done a really good job given the situation, but you don’t want to point out you’ve missed this, this, and this. But you kind of want to share some things that you were thinking while you were observing.

Students began to openly and more frequently express their pride when the team worked well during a simulation scenario and collectively took responsibility when scenarios did not go to plan. As the course progressed, students became less nervous about not knowing what they would face in simulation scenarios and performing in front of peers and staff. Students became more focused on reassessing their knowledge base and ideas and discerning how to work in acute situations with their peers – no matter their profession - for a good ‘patient’ care outcome. By the end of the week, we started to see evidence of a solidified new state whereby students’ self-conception included an interprofessional dimension.

## Discussion

Our findings suggest that students attending UIPCW acquired more than new knowledge; students were also challenged to view their professional selves in new ways. Our data also highlights that the concept of liminality can help educators better understand the value of interprofessional activities at the pre-registration stages of health professional education. Students at UIPCW can be seen unravelling, discarding, and rewriting their own professional scripts. What emerges from our data is that student cognitive scripts about their own and other professions were easily and quickly rewritten through ritualised and repeated learning activities (such as the structured communication tool ISBAR and structured debriefs). Student participants in the UIPCW study arrived with pre-existing and primarily uniprofessional identities yet by the end of the week we start to see the emergence of a developing “interprofessional” state. Our data draws attention to the complex role of formative educational experiences such as UIPCW on student identity construction. Our data supports Khalili et al’s [14] assertion that socialization is an important component to the development of an interprofessional identity (as an identity that sits alongside a uniprofessional identity). Our findings also highlight that IPE occurring during pre-registration training may contribute in a small way to the flattening of hierarchies between professional groups because, as liminals, they are gaining an appreciation for other professions.

By the end of the course, students demonstrated shifts in professional identity construction to emphasise interprofessional and team dimensions. This shift signalled an evolving conception of relatedness between professions. Over four days – a relatively short period when considered within the context of a professional degree – students demonstrated shifts in their identity construction from individuals - with unique scopes of practice and specialised professional knowledge - to team members with shared and effective communication strategies and care goals.

This shift, we suggest, was successfully supported through ritual. Rituals can be defined as prescribed formal patterns of cultural behaviour, and they are often repeated and hold predictable rhythms ([53], p. 186). These routines and rituals serve to secure order within a broader social structure so that those within a particular culture can understand what is expected of them. Swidler [54] asserts, “when people are learning new ways of organising individual and collective action, practicing unfamiliar habits until they become familiar, then doctrine, symbol and ritual directly shape action” (p. 278). Regarding developing professional and interprofessional identities, we suggest that during UIPCW students experience new rites of passage that effectively transition students to become interprofessional members of broader communities of practice. Simulation, for instance, is a highly ritualised form of learning and forms an important component of UIPCW. For example, there are rituals for communicating in structured ways. There are also rituals associated with performing ways to call for help and when to call for help – students learn through interprofessional simulations when and how to call for help in routine ways. Our data highlights that it is during simulation activities that we see conceptions of the professional self shift more readily. Thus, on UIPCW we see that simulation – supported by small group learning activities and social activities –disrupts preconceived ideas about roles and can assist students to form new cognitive models of what it means to be and act as a healthcare professional.

Similarly, Land et al. [55] suggest that threshold concepts can help us conceptualise transformative learning settings. Threshold concepts are difficult and important concepts or practices that students need to master in their professional training to progress. They can act as a portal through which a new perspective opens for a learner. These learning moments can transition a student to a new level of insight and reformulate the learner’s subjectivity [36]. We observed that many of these learning moments were instigated by the presence of a professional or student from another discipline. Learning alongside other professional groups saw students shift their understanding of their role as a health professional and the beginnings of shared interprofessional identity. Thus, ritualised IP learning differs from similar uniprofessional learning because notions of the professional self are constantly and intensively called into question by the presence of the clinical other. As our data highlights, this shared identity discourages biomedical gazing and emphasises a team approach to care.

Can these small shifts in identity formation that we observed during a four-day course create sustainable change towards increased effective IP? Are there other experiences that can support the development of an interprofessional identity during pre-registration training? How are these identities then sustained and solidified as students move into clinical environments? Longitudinal research could answer these questions, and we encourage researchers to look to longitudinal or control study methods to help complete this important picture of identity formation and IP among healthcare professionals.

## Strengths and limitations

In medical and health sciences education literature ethnography is an underutilised approach. In this study we have drawn together a range of data types – direct observational, self-report, photographic, film, illustrative – to understand and exemplify a complex process and this is a strength of the study. The focused ethnography method meant that we focused solely on student experiences during a four-day course. There are likely to be many other influences or interactions outside these four days that contribute to student identity formation. We also acknowledge that approximately one-fourth of students chose to decline to participate in this study, which could impact our findings. A possible reason for non-participation might be that students may have felt like participating in the research might have added additional pressure to perform under already stressful circumstances. The second author has prior immersive experience with UIPCW as faculty, and this may have informed her interpretation of the data. However, three of the ethnographic observers (including the first author) had no prior experience of UIPCW, so this likely minimises such potential influence over the findings reported here. The researchers used multiple methods of triangulation to improve the credibility and trustworthiness of the analytic process and to scrutinise the findings outlined in this article [44]. We used a range of data collection methods such as observational field notes, participant observations and ethnographic film. We also observed participants in various educational settings and social settings, including classrooms, simulations, debriefing sessions and as participants interacted during short breaks. The researchers followed up with participants on breaks to ensure that what they had written reflected their experiences. Ethnographic observers also discussed the finding between themselves during field observations and data collation and analysis. Both authors were familiar with the concept of liminality before conducting the research, but its relevance only became apparent during the analytical process. The authors also tested several relevant social theories for interpreting the data before establishing the theoretical lens outlined in this article.

## Conclusion

The UIPCW course represents a unique liminal space where students’ conception of what it means to be professional is called into question, narratives about what it means to be a healthcare professional are challenged and disrupted, and students emerge with a new understanding of the professional self. Interprofessional aspects of UIPCW disrupt the process of biomedical gazing as students are challenged to ‘become’ effective team members alongside students and disciplinary specialists from various disciplines. This study suggests that the identities of liminals shift frequently and easily, perhaps more so than in the later stages of clinical training. Our data highlights that developing an interprofessional identity in the early stages of training allows students to understand their own and others’ scopes of practice more comprehensively and may contribute to the flattening of hierarchies in the clinical setting. Encouraging students to construct a strong interprofessional aspect to their professional identity in the early stages of their training may also offset detrimental aspects of hierarchies of power in the clinical setting and promote a more holistic and rich collaboration between healthcare professionals.

## Data Availability

The data that support the findings of this study are available on request from the corresponding author. The data are not publicly available due to privacy or ethical restrictions.

## Abbreviations

IPE: Interprofessional Education
UIPCW: Urgent and Immediate Patient Care Week
IPEC: Interprofessional Education Collaborative
IP: Interprofessional practice
CAIPE: The Centre for the Advancement of Interprofessional Education (CAIPE)
